# Vestibular rehabilitation with biofeedback in patients with central imbalance

**DOI:** 10.1590/S1808-86942011000300014

**Published:** 2015-10-19

**Authors:** Roseli Saraiva Moreira Bittar, Camila de Giacomo Carneiro Barros

**Affiliations:** 1 PhD in Medicine, Assistant physician in the Neurotology Department - HCFMUSP (Medical School of the University of São Paulo); 2 MSc in Medicine, Professor - FAEPA - Medical School of Ribeirão Preto– SP; Neurotology Department - Medical School - University of São Paulo

**Keywords:** stroke, rehabilitation, dizziness, postural balance

## Abstract

Central Nervous System disorders may cause important functional unbalance in the maintenance of balance and posture. There is no effective rehabilitation for these symptoms until now.

**Objective:**

The aim of this paper is to evaluate the use of tongue electrotactile stimulation on patients with central imbalance using BrainPort.

**Materials and Methods:**

This is a prospective case series study. We evaluated 8 patients with central imbalance, 6 men and 2 women, with mean age of 67.75 years. The patients were submitted to Computed Dynamic Posturography (CDP) and then received 18 sessions of electrotactile stimulation by BrainPort® device for 20 minutes, twice a day. Then they were submitted to a new CDP test and to a self-perception scale to assess symptom remission, partial improvement and no improvement at all.

**Results:**

75% of the patients reported being more stable. There was no improvement in the balance control of the mass center in these patients.

**Conclusion:**

The patients were able to use the electrotactile stimulus to improve their balance control.

## INTRODUCTION

Balance control is one of the most complex sensorimotor functions in our bodies. For its perfect performance it is necessary that vestibular, visual and proprioceptive information be understood and integrated by the central nervous system. The performance and maintenance of anti-gravitational balance and proper responses to balance disorders depend on the very accuracy of this information and its integration^1^. Vestibular information is essential for our proper positioning in the tridimensional space. Spatial perception and that of the relationship of moving objects, visual and memory integration also depend on vestibular functions^2^, ^3^. Vestibular afferent and efferent signals also act helping coordinate movements, internal orientation, muscle tone and body alignment^4^. In the absence of a functional vestibular system, the central nervous system (CNS) has great difficulty to properly integrate the information coming from other afferent, visual and proprioceptive systems.

CNS lesions cause sensitive, motor and cognitive losses which peak with the involvement of balance control - a key component of the mobility problems experienced by patients who had a Cerebral Vascular Episode (stroke). Difficulties include intense instability, body weight asymmetrical distribution, and a reduction in the person's ability to maintain posture alignment. Thus, balance, gait and mobility represent the intervention targets in the rehabilitation process of these patients. Among possible approaches in these cases we have motor therapies, neurophysiological approaches, the force platform, repetitive training and biofeedback^5^.

Lesions arising from strokes may affect different areas of the CNS, including the cerebellum - a key component in the coordination and integration between afferent and efferent I/O - which make up balance. Concerning ataxia, there are very few studies which report on the effects of rehabilitation treatment; nonetheless, there are indications that the training program brings about benefits - even when it does not yield definitive results and complete improvement^6^.

In Parkinson's disease (PD), the most disabling symptoms involve gait and balance problems. Major clinical significance has been attributed to the falls suffered by PD patients, which are caused by independent, but coexisting, mechanisms: balance instability, difficulties with transfers (change in position), gait changes and orthostatic syncope^7^. Treatment is based on drug therapy, neurosurgery and physical therapy, but none of these options is efficient when used alone.

In searching for feasible alternatives to reestablish the neuronal circuits compromised by diseases which affect the CNS, recent studies have shown the efficacy of alternative mechanisms (*balance devices)* in reestablishing body balance. This device is able to inform the CNS about head position - information which usually comes from the vestibular system, through tongue electrotactile stimulation^8^. In order for the brain to properly interpret the information from the sensorial substitution equipment, it is not necessary that it is presented the same way as the natural sensorial system. It is only needed that the information be captured and coded in action potentials - which this time comes from an alternative channel and not from the original physiological sensory system - in this case: the vestibular system. With training, the brain learns to properly interpret information and use it similarly to the perception of data by the original system^9^.

After satisfactory results using the biofeedback and electrotactile equipment (BrainPort®) in patients with bilateral vestibular arreflexia^10^, we thought about checking its applicability in patients with central balance disorder. The goal of using biofeedback would be to stimulate the alternative neural pathways which could help maintain balance.

## OBJECTIVE

To describe the impact the tongue electrotactile stimulation has on the balance control of patients who have CNS dysfunctions and did not obtain satisfactory results with vestibular rehabilitation and/or physical therapy.

## MATERIALS AND METHODS

This study was previously considered and approved by the Ethics in Research Committee - CAPPesq from the Clinical Board of our Institution (number 1366/06). This is a case-series study type.

The participants were selected from our Neurotology Ward, to where they went looking for treatment for their balance disorders. They were all fully briefed on the procedure and agreed to sign the informed consent form.

We included those individuals who had balance disorders secondary to CNS dysfunctions, previously submitted to physical therapy and vestibular rehabilitation (VR) without satisfactory clinical improvement. We took off the study those patients who had mouth and tongue lesions; smokers; those with electrical implants such as heart pace-makers or those with orthopedic lesions in their lower limbs. After clinical assessment and balance disorder characterization the patients were submitted to a quantitative assessment of their balance disorder by means of computerized dynamic posturography (CDP) twice - before and after electrotactile stimulation therapy. The assessment protocol utilized was the Sensorial Integration Test (SIT). We carried out 18 electro stimulation sessions, lasting 20 minutes each: twice a day, thrice a week in alternate days, in a total of 3 weeks. As CDP variables we considered C5 (condition 5), C6 (condition 6) and BI (Final Balance Index), before and after treatment. We recorded body oscillation during testing before and after electrotactile stimulation.

The electrotactile stimulation was done on the tongue dorsum by intraoral equipment made up of a plate of electrodes with 10 rows with 10 stimulation points - BrainPort®. The plate is connected to one accelerometer which identifies the stimulus movement throughout its surface, following the patient's head movements. The patient is trained to centralize the tongue stimulus and thus keep oneself straight. The detailed description of the equipment and that of the procedure can be found in reference # 11^11^.

After finishing with the electrotactile stimulation training sessions, the patients answered a visual-analogue scale (VAS) with the aim of assessing the clinical impact of the training. Symptom self-perception after training was assessed according to three criteria:

• Remission (R): corresponding to 100% of symptom relief.
• Partial improvement (PI): from 50 to 90% of symptom improvement.
• No improvement (NI): less than 50% of symptom improvement.

Table 1 depicts the body balance disorder etiologies, age and gender of the patients who were studied.

**Table 1:** Patient distribution according to age, gender and etiology.

PATIENT	GENDER	AGE	ETIOLOGY
1	M	69	HI
2	M	75	STROKE
3	M	67	STROKE
4	M	70	PARKINSON
5	F	55	ATAXIA
6	M	66	CEREBELLAR TB
7	M	71	STROKE
8	F	69	STROKE

HI: Head Injury; TB: tuberculosis.

CDP objective measures (C5, C6 and BI) before and after training were compared by means of the Student T test.

## RESULTS

Description of the cases assessed and their progress:

Patient 1: **AMP** - male, 69 years. A past episode of fall with head injury and prolonged brain anoxia. His main complaint was balance disorder and instability. He had ataxic gait and started training with difficulty to keep his eyes shut, even on a stable surface. After six sessions (one week), he was able to keep his eyes shut on an unstable surface (foam) during the 20 minutes he was using the device. He was very pleased with the treatment and had better stability and greater confidence, he also felt safer to walk.
Patient 2: **JCR -** male, 75 years. Trunk stroke with hemiplegia. His main complaint was balance disorder, without other sequelae. He started training without being able to keep his eyes shut on a stable surface. After 12 sessions (two weeks), he could remain for short times with his eyes shut on an unstable surface; at the end of 18 training sessions he was able to remain for 20 minutes of training with his eyes shut on an unstable surface (foam). At the end of the training period he felt safer during gait, however without complete remission of his balance disorder.
Patient 3: **ACL** - male, 67 years, trunk stroke with hemiplegia. This patient had mainly instability and gait disorder especially in sudden movements used to turn the body. He had mild gait stiffness and step mismatch. In the beginning of treatment he was unable to keep his eyes shut on an unstable surface. With treatment he developed a mild balance improvement in performing the task. At the end of the 18 sessions he had better balance and better gait performance. He maintained his difficulties with sudden movements, despite a mild improvement.
Patient 4: **ND** - male, 70 years old, Parkinson's disease. Classical complaints of stiffness, bradykinesia and tremor. He did not have severe balance disorder at the onset of training. After the third session he was able to keep his eyes open on an unstable surface (foam) and developed excellent body command as the training challenges increased. At the end of the 18 training sessions, the patient felt safer and more stable to walk. Tremor and muscle stiffness remained - the symptoms which bothered him the most. We consider his improvement as partial.
Patient 5: **DJ** - female, 55 years old, cerebellar ataxia. Her main complaints were associated with gait and balance. She was visually challenged, she was unable to stand on an unstable surface with her eyes shut. In her last 4 sessions she was able to keep her eyes shut for short periods of time during the 20 minute-session of treatment. She did not perceive any improvement, although her CDP showed an improvement in performance concerning the many conditions she had.
Patient 6: **RH -** male, 66 years, Cerebellar TB. Complaining of unbalance type of dizziness, triggered by visual stimuli. Symptoms happening mainly in areas of excessive visual stimuli, such as busy streets and supermarkets. Since the beginning of his training he was able to perform the most difficult task which is to keep eyes shut when on an unstable surface. At the end of treatment he did not report subjective improvement. His CDP performance remained unaltered.
Patient 7: **AM**, male, 71 years, trunk stroke. Complaining of lack of balance and instability. Little difficulty in keeping eyes shut on an unstable surface. In the first week he managed to execute the most difficult task during the 20 minutes. At the end of the sessions he reported subjective improvement, although his CDP performance remained unaltered. We noticed a mass center alignment upon CDP, in agreement with his self-perception regarding treatment outcome.
Patient 8: **MD -** female, 69 years, trunk stroke, 1 year of physical therapy and VR. She complained of instability and unbalance, she used a cane on one side and she held onto another person on the other side in order to walk. She was unable to keep her eyes shut on a firm surface. With ten training sessions she was able to walk with the cane, without needing to hold onto another person. At the end of the 18 sessions she managed to keep her eyes shut on a foam surface, although with some body oscillation. She considered her result as an improvement in her static balance and a safer gait. At the end of treatment she complained of fatigue in her lower limbs and could not stand up for long periods of time because of weakness on her affected lower limbs.

Of the 8 patients treated, 6 (75%) had subjective partial improvement - between 50% and 70% according to the VAS. Also according to the VAS, two individuals did not reach 50% of balance improvement after treatment. There were no reports of worsening and we also did not observe adverse effects.

When we assessed CDP results, the 6 patients who reported subjective improvement also had an increase in BI - objective measure of balance assessment (Table 2). On the same Chart 1, we notice that patients 2, 3, 4 and 8 had a drop in their CDP test situations which were carried out on an unstable surface (C5 and C6) both before and after treatment. Patient number 4 was the only one who reported an improvement in his performance on condition 5 - unstable surface without visual support.

**Table 2:** Mean values of the conditions assessed and balance indexes on CDP before and after treatment.

PATIENT	C5		C6		BI	
	PRE	POST	PRE	POST	PRE	POST
1	8	55	46	31	46	61
2	0	0	0	0	40	46
3	0	15	0	0	49	53
4	14	68	67	75	68	81
5	0	0	0	0	33	39
6	53	54	42	41	69	69
7	34	17	61	52	61	61
8	0	10	0	0	29	33

0: means fall

There was no significant difference when we analyzed the mean values obtained before and after treatment on C5, C6 and BI. VAS information are in agreement with BI findings, which reflect a mild improvement in those individuals who were studied.

**Figure 1:**
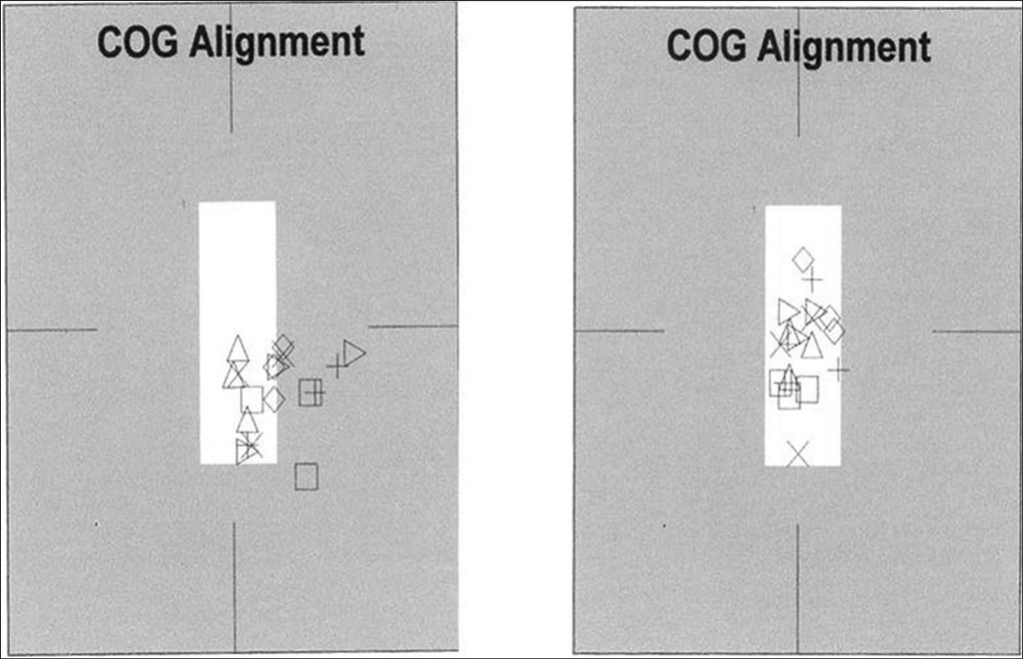
Documenting body oscillation in patient 6 before and after the electrotactile stimulation. The geometric figures observed correspond to mass center positioning of each one of the CDP tests.

Concerning patients 6 and 7, there were no improvements in the assessed parameters, or even of the VAS. We did notice, however, that both had a better positioning of their mass centers and less body oscillation after treatment (Figure 1).

## DISCUSSION

It is estimated that the percentage of patients who have CNS diseases which affect balance varied between 7% and 45% in specialized tertiary services in the USA. Unfortunately, very little is known about the effects of rehabilitation treatments in these cases, especially when we consider the large number of conditions which can be responsible for central balance disorders^12^. Central-origin body balance has a negative impact on the quality of life of patients for a number of reasons, from the very feeling of unbalance, all the way to gait involvement. Falls play a fundamental role in these diseases, because of muscle weakness, visual impairment, the use of multiple drugs, neuro-cardiovascular instability and also environmental factors.

Our sample has patients with body unbalance of different etiologies of central origin, previously submitted to VR and who had limited results. In fact, in a previous retrospective study, we noticed that our series of patients, to whom VR was indicated, is made up of 13.5% of central etiologies. These patients, when submitted to rehabilitation by conventional methods did not have satisfactory results in 37.5% of the times^13^.

Some alternative treatment approaches have been used in patients with bilateral vestibular dysfunctions with the goal of improving their performance in balance and posture control. Among these approaches is the use of non-implantable devices which work as sensorial substitutes. These devices provide a neurofeedback, in other words, the feedback of the movement information which is turned into a stimulus, different from the original stimulus coming from the vestibular stimulation. These alternative stimuli, which inform about body movement, may be auditory, galvanic or vibro-tactile^14^, ^15^, ^16^. Among the organs which can work as receptors for these stimuli we have the tongue - a rich sensorial terminal connected to important structures in the brain stem. The stimulation of its terminals is able to active effective mechanisms in the control of posture, although the network of neural distribution in which these information travel are yet to be totally unveilled^17^. The idea is to provide alternative information which would guide postural correction by means of ancillary devices which aim at replacing the compromised vestibular information. Besides the process of body movement recognition, the tongue electrotactile biofeedback promotes another interesting effect: patients with bilateral vestibular areflexia who were treated by the method could retain the learning acquired after stopping using the device^11^. Since we noticed beneficial results from the device without adverse effects, we decided to apply this technology to patients with central balance disorders. This technique is already successfully used in other world centers for the treatment of people diagnosed with CNS diseases^18^. Our goal was to offer some additional improvement besides the one obtained from conventional VR, which is very limited in these cases.

CDP is the tool-of-choice to assess the results obtained from training, because the mobile platform situation (C5) mimics exactly the straight posture with the eyes closed on foam - a training situation which is the most difficult for patients. It is also situation 5 which simulates and documents the vestibular performance, which when not adequate ends up producing a fall. Therefore, the fall is the posturographic situation which translates postural maintenance failure. As seen on Chart 2, most of the patients had falls in these situations before treatment by biofeedback. This observation tells us about an important involvement of posture that these individuals had, upon treatment onset: the failure in postural maintenance under sensorial conflict situations. The fall observed is the immediate consequence of the stabilization difficulty of the mass center. Even without a visible improvement in the final indices obtained from the CDP analysis, the best performance as to body stability, a fundamental factor in the prevention of falls, was noticed in all the individuals (Figure 1). We believe that the increase in sample would be able to refine the results obtained, turning this observation into statistical significance.

Among the patients studied, patient 4, with Parkinson's disease was the one who presented the highest improvement indices in CDP. Although such improvement was seen, the patient's major complaint was the typical tremor of Parkinson's and, therefore, the improvement was deemed partial. The worst results from self-perception were observed in the patients with cerebellar involvement. Such occurrence is not unusual, because the cerebellum is responsible for integrating and coordinating the afferent and efferent signals associated with body balance. In fact, in our clinical experience, the diseases which affect the cerebellum are the same ones which yield the worst results in body balance rehabilitation.

The results suggest that some patients were able to use the electrotactile stimulation as ancillary information in the perception of head position in relation to the vertical gravitational orientation and use it to maintain posture. It is also valid to consider the subjective improvement of the patients, according to the VAS, because instrumental tests, such as CDP are not able to inform about the impression of the patients as to the dynamic performance, such as gait, for example. The statistical values which do not show differences between the results before and after treatment corroborate this idea. In this study we did not remove many of the drugs being used and some of them may have had a negative impact on balance recovery because they are CNS sedatives and necessary for these patients to avoid convulsion, for instance. Although the results have been restrict and encompass a small number of patients, we can see the likelihood of using a new resource to manage individuals who have had very limited possibilities of improvement so far. We also believe that some mild changes in the treatment protocol, such as daily sessions with longer periods may provide additional benefits to the patients. Comparative studies are still needed to assess not only the clinical benefits of the device in the different diagnoses, but also to establish the necessary treatment time for a more effective recovery.

## CONCLUSION

Some patients with central balance disorders were able to use the electrotactile information to tune up their postural control. Since this was not a controlled study, we cannot establish the type and reach of these changes, not even those which happened during treatment with the electrotactile biofeedback device. Notwithstanding, in some patients it was possible to observe an objective improvement in balance control - confirmed by the CDP results - exceeding the results obtained so far with physical therapy and conventional VR.

## ACKNOWLEDGEMENTS

We thank FAPESP for their financial support (process 08/54324-3)
